# Dissecting an adiposity locus with an arsenal of genomics

**DOI:** 10.1186/s13059-018-1460-y

**Published:** 2018-06-07

**Authors:** Kim Lorenz, Benjamin F. Voight

**Affiliations:** Department of Systems Pharmacology and Translational Therapeutics and Department of Genetics, University of Pennsylvania-Perelman School of Medicine, Philadelphia, PA 19104 USA

## Abstract

We discuss a recent study that has identified and validated the link between a type-2 diabetes (T2D) association and human adipose biology by means of *KLF14* gene expression. In addition to being maternally imprinted, the contributed risk at this locus is greater in female carriers.

## Introduction

Extracting the causal genes, intermediate traits, and molecular phenotypes relevant in individual subjects from each of the thousands of loci associated with complex diseases remains a daunting challenge. In a recent study, Small et al. [1] have begun to fill in the biological blanks between risk allele and phenotype for a type-2 diabetes (T2D) locus, identifying relevant changes to adipose tissue in genotyped biobank patient samples. Their focus is *KLF14*—a maternally imprinted transcription factor known to be a trans regulator of adipose gene expression and linked to established loci for T2D, heart disease, and hypercholesterolemia. The T2D- and high-density cholesterol-associated variants in the region are both cis- and trans-expression quantitative trait loci (eQTLs) for *KLF14* in adipose [2, 3], making it the leading causal gene candidate. But, beyond these tissue expression patterns, very little is known about how *KLF14* might affect T2D, other metabolic traits, and cellular phenotypes.

## Confirming the basics

The authors begin by confirming the initial molecular phenotypes in the region, and explore the potential to identify a causal variant culprit contributing to the underlying association. The previously observed cis and trans expression network was verified by means of TwinsUK RNA-sequencing data and confirmed in three additional cohorts. However, finding the causal variant was more challenging—the seemingly ancestry-specific (European) association precluded attempts to refine the association through trans-ethnic fine-mapping approaches. Nevertheless, the authors were able to fine-tune this association by using functional regulatory features from ENCODE and the Epigenome Roadmap, coupled with methylation profiling data, again from TwinsUK participants. These sets of data pointed to an adipose-specific enhancer element within the T2D credible single-nucleotide polymorphism (SNP) set that shows the expected maternally imprinted methylation pattern and corresponding *KLF14* expression change.

## Expanding the *KLF14* trans network

Given that *KLF14* serves as a trans-regulator, Small and colleagues next sought to expand the tissue-specific trans network using the above RNA-sequencing data. This identified 385 trans genes having both positive and negative effects with regards to the T2D risk allele. After confirming that these results were adipose limited, they used mediation analysis, enrichment of chromatin immunoprecipitation sequencing (ChIP-seq) peaks, and enrichment of the KLF14 motif to support the hypothesis that *KLF14* itself is responsible for these trans network changes. Nearly half of the identified genes contain a proposed KLF14 binding motif, and these genes are enriched for relevant ToppGene [4] annotations. More excitingly, they found several genes in the trans network already known to affect metabolic syndrome phenotypes: *SLC2A4* (glucose uptake) and *IDE* (insulin degradation), as well as the T2D-associated genes *STARD10*, *C6orf57*, and *CDK2AP1*. This suggests that the *KLF14* risk allele likely affects T2D and related phenotypes through changes to multiple genes and biological pathways across the trans network.

## Sexual dimorphism of *KLF14* variation on human metabolic traits

Given previous indications of metabolic syndrome-related phenotypes related to the *KLF14* locus and its imprinted status, the authors reanalyzed the related network of traits, including sex-stratified analyses. Broadly, they found metabolic trait associations with pronounced effects in women relative to men, strikingly for measurements of fasting insulin levels and hip circumference, but also non-trivially for T2D risk. Considering the parent-of-origin and sex effects at the locus, they suggest a 30% T2D risk increase for women that inherit the risk allele from their own mothers. By leveraging TwinsUK data, the authors also noted a change in the fat distribution in females, with the risk allele affecting the ratio of android to gynoid fat, but not total fat amount. These data ultimately suggest that reduction of expression of *KLF14* in adipose contributes to an insulin-resistant, T2D-predisposing phenotype (particularly in women), characterized by a shift of stores of adipose from abdominal to gynoid depots.

## Looking to mouse models for answers

Mouse *Klf14* knockouts were broadly consistent with the identified genome-wide association study (GWAS) results, showing lower high-density lipoprotein (HDL), higher triglycerides, and impaired glucose and insulin tolerance. However, the mouse models did not fully and completely recapitulate the female-specific impact of the risk allele, possibly owing to complete removal of gene product or differences in mouse and human adipose biology. Indeed, comparing genes between the trans network identified in human with differentially expressed genes identified by RNA-sequencing of subcutaneous fat obtained from the *Klf14* mouse knockouts demonstrated significant overlap across the network, but only a fraction of that network was shared. This might not be a complete surprise, given the complexity of mechanism at this locus, and lack of strong differences across male and female mice.

## Insights gleaned from genotype-based biobank queries

Lacking mouse models consistent with the human results, the authors then turned to primary adipose samples to measure phenotypes related to the *KLF14* genotype. They first assessed *KLF14* expression in primary preadipocytes differentiated in culture, finding that *KLF14* is consistently expressed higher in females, consistent with the sex-specific effects observed in GWAS. Using the Oxford BioBank, they were able to perform genotype-targeted phenotyping of primary adipose tissues from males and females homozygous for the risk or control alleles for a number of relevant adipose-biology phenotypes, ranging from accumulation of metabolites to cellular morphology. Among several observations of note, they reported female-specific defects in glucose uptake, a reduction in lipogenesis, increase in proliferation, and increase in adipocyte size in risk-allele carriers compared with controls. The authors showed consistent effects with short hairpin RNA (shRNA) knockdowns of *KLF14* and across multiple sampling cohorts, clearly replicating effects across multiple assays.

## Concluding remarks

Overall, the authors propose a clear and plausible mechanism for T2D risk at the *KLF14* locus—the risk allele reduces *KLF14* expression, affecting a network of downstream genes in adipose, which leads to glucose uptake-deficient adipocytes that store fewer lipids, therefore increasing their insulin resistance and T2D risk. They identify a possible enhancer relevant to the maternal imprinting, several plausible gene targets of KLF14, and a sex-specific expression difference that is probably at least partially responsible for the sex-specific effects. All of this complex biology underlies a single GWAS locus, and will directly impact treatment possibilities—based in this case both on imprinting and sex-specific biology.

As more and larger biobanks become increasingly available, we can anticipate that genotype-based phenotypic mining of these data will become increasingly mainstream for characterization of the underlying genetic associations when a relevant tissue or cell type is known or suspected. This seems to be especially true in adipose biology, where tissues are relatively easily accessible, primary lines can be established routinely, and where an array of molecular and cellular phenotyping options is readily available.

## Abbreviations

ChIP-seq: Chromatin immunoprecipitation sequencing
eQTL: Expression quantitative trait locus
GWAS: Genome-wide association study
HDL: High-density lipoprotein
SNP: Single-nucleotide polymorphism
T2D: Type-2 diabetes
